# Supplementary material for: Are carbon emissions trading and green financial instruments synergistic? -Comprehensive quantitative research based on content analysis
Source: PLoS One. 2024 Mar 7;19(3):e0298601. doi: 10.1371/journal.pone.0298601 (PMC10919601; doi:10.1371/journal.pone.0298601)
Supplement: S1 Data — (ZIP) [file pone.0298601.s001.zip › Data/地级市Z_F工作报告.zip!/┴╔─■/╕o╦│╩╨/└┤╫╘╣1⁄2╓┌║┼í╛┬φ┐╦╩2╛▌═°í┐_2013.docx]

各位代表：

　　现在，我代表市政府向大会报告工作，请予审议，并请市政协各位委员提出意见和建议。

　　　一、本届政府暨2012年工作回顾

　　本届政府的五年极不平凡。五年来，在市委的正确领导下，在市人大和市政协的监督支持下，我们团结带领全市人民，积极应对各种风险挑战，坚定不移地以经济建设为中心，践行以人为本、科学发展、建设幸福抚顺的执政理念，大力推进经济建设、文化建设、社会建设和生态文明建设，城市综合实力大幅提升，人民生活水平显著提高、幸福感明显增强。

　　2012年预计完成地区生产总值1230亿元，公共财政预算收入130亿元，城市居民人均可支配收入20960元，农民人均纯收入10185元，分别是2007年的2.2倍、2.9倍、1.9倍和2.1倍。

　　五年来，我们全力推进经济结构战略性调整，区域发展更加协调,产业结构日趋合理。“两城两带”、全域发展、多点支撑的发展战略深入实施，县区第一轮三年倍增计划全面完成，沈抚新城成为沈阳经济区的先导区和示范区，石化新城纳入沈阳经济区新城新市镇发展规划。农业综合生产能力明显提高，工业核心竞争力显著增强，以旅游为牵动的服务业加速发展。

　　五年来，我们坚持改革开放，国企改革画上圆满句号，一批老企业焕发生机；工业产业集群、服务业城市综合体、农产品深加工等一批项目成为新的经济增长点；累计全社会固定资产投资3330亿元，是上个五年的4倍；向上争取资金超过300亿元，抚顺成为国家资源枯竭型城市转型试点市和能源装备高新技术产业化基地。

　　五年来，我们一手抓基础设施建设，一手抓环境面貌改善，城乡统筹发展迈上新台阶。高标准绿化、亮化、美化、净化城市，基本建成“五点一线”浑河水系景观，城区主干街路全面改造一新，“摸黑路”成为历史，创造了绿水工程、生命之环、月牙岛生态公园、临时替代热源等城建史上的奇迹。县城和重点小城镇规划建设全面展开，率先在全国试点完成400个村环境连片综合整治，二级以上公路比重跃居全省第一，新农村建设取得可喜成绩。

　　五年来，我们在改善民生和创新管理中加强社会建设，集中连片棚户区改造圆了32万居民的住楼梦，保证了企业离退休人员养老金按时足额发放，实现了新型农村养老保险和医疗保险全覆盖。群众性文化体育活动蓬勃开展，雷锋城建设取得阶段性成果，抚顺连续五年荣获省平安建设先进市称号。

　　五年来，我们全面加强政府的作风建设和队伍建设，先后开展“三讲一鼓劲”、“三抓实、三走进”等活动，锤炼了一支懂经济、善管理、有作为的干部队伍，涌现了一批素质高、能力强、富有社会责任感的企业家，为加快发展奠定了坚实的人才基础。政府工作越来越得到群众的理解和支持，凝心聚力、共建幸福抚顺的内生动力已经生成。

　　回首征途，全市人民同甘共苦、风雨同舟所走过的五年，是经济快速发展、城乡环境发生巨变的五年，是改革开放力度加大、民主法制建设迈出坚实步伐的五年，是人民生活水平迅速提高、城市凝聚力和竞争力明显增强的五年，更是抚顺人重树自信、倍感自豪的五年。五年的发展成就令人振奋，五年的思路举措弥足珍贵。只要我们一以贯之地坚持下去，就一定能够战胜各种艰难险阻，就一定能够再创佳绩、再铸辉煌！

　　各位代表，刚刚过去的2012年，在外部经济环境十分严峻复杂的情况下，我们在发展中把握机遇，在困境中奋发有为，向建设幸福抚顺又迈出了更加坚实的一步。

　　（一） 应对危机成效显著

　　准确判断形势，坚持稳增长与调结构并重，抓工业、促三产、保投资，抚顺始终站在全省经济企稳回升的前列。

　　工业逆势而上，企业核心竞争力显著增强。老企业稳定增长，工业用电量增速稳居全省第一，预计完成规模以上工业增加值610亿元、增长13%。12个产业集群加速发展，新项目增添后劲，完成投资500亿元、增长30%，133个亿元以上重大工业项目全部开工建设，51个项目竣工投产，投资和投产数量均创历史最好水平。以香港太平洋工业城为代表的中小企业群迅速崛起，实现工业增加值432亿元。

　　服务业发展迅猛。预计完成增加值410亿元、增长11.5%，税收占地税收入57%；就业人数达到53万人，占全市就业总量46%。旅游项目建设取得新进展，成功举办满族风情节、冰雪节、戏水节，首次推出“五一”市民免票游景区、“十一”百万大礼送游客活动，全年游客接待量和旅游收入均创历史新高。商贸业提质提速，一批城市综合体项目主体完工，在建商业面积400多万平方米，新抚中央商务区初具形象，榆林市场群基础设施开工建设。房地产开发投资额、商品房销售额均增长15%。金融、交通运输和仓储业等生产性服务业比重提高4.8个百分点。

　　投资力度进一步加大。强化主题概念和“一把手”招商，鼓励本地投资，吸引外来投资，域外引资到位额700亿元、增长46%；向上争取资金85亿元；贷款余额552亿元、增长19.5%；全社会固定资产投资增速位居全省第二。

　　（二） 两座新城快速发展

　　坚持给空间、给权力、给财力，促进新城迅速崛起。

　　沈抚新城发展突出一个“新”字，管理体制创新试验方案获省政府批复；基础设施和城市功能进一步完善，沈抚同城化取得新进展，9条道路与沈阳对接，东北育才实验学校学生拥有沈抚双学籍。“一带三区”空间布局已经形成，产业区新投产10亿元以上项目7个，新增同行业一流核心技术企业9家；核心区基本建成总部基地19家和金风湾、玉露潭水系，完成文化中心、研发中心主体建设；生态区巴厘岛水世界、皇家海洋主题乐园游人如潮。沈抚新城综合经济实力稳居全省新城新市镇前列。

　　石化新城发展突出一个“变”字，产业发展、城市更新和民生改善同步推进。以延伸石化产业链为重点的新型工业化产业体系初步建成，总投资159亿元、41个重点项目落户高新技术产业园区，大力推进以商业、卫生、教育等城市功能完善和路网、管网基础设施建设为重点的城市更新改造，昔日老旧落后的工矿区开始脱胎换骨，踏上发展振兴的新征程。

　　（三）县区活力进一步迸发

　　 大力实施县区经济倍增计划，强县壮区迈出坚实步伐。

　　县域经济加速发展，粮食产量实现七连增，农民人均纯收入超万元。清原县、新宾县地区生产总值和固定资产投资双双突破百亿元，抚顺县主要经济指标增速位居全省前列。中药材、食用菌规模分别位居全省第一和第三，特色产业占农业比重超过50%。 

以服务业为重点的城区经济快速发展，城市更新步伐加快，新抚区经济强区地位继续巩固，商业中心的影响力进一步扩大；顺城区一批重大商贸项目落地建设，城东高端商住新区初具规模；望花区拆迁改造30万平方米，工业老区展现新姿。

　　（四）城乡面貌日新月异

　　实施六大工程，努力建设山清水秀、生态宜居城市，全省绿化、亮化、美化、净化工作现场会首次在抚顺召开。

　　绿水工程超额完成。在全省率先启动绿水工程，关闭污染企业470家，治理农村中小河道260公里，浑河和13条支流河全部得到整治美化，全市污水处理能力达到100%，浑河干流和支流水质基本达到功能区标准，污水直排成为历史，水清、岸绿、景美成为现实。

　　青山工程扎实推进。查处涉林案件591起，完成小开荒还林7万亩、退坡还林1.45万亩，生态治理闭坑矿山1250亩、生产矿山717亩，绿化覆盖公路沿线破损山体690处，东露天矿、红透山矿、傲牛铁矿被授予“国家级绿色矿山”称号。

　　蓝天工程启动实施。建成并稳定运转化工、冶金行业脱硫项目7个，关闭污染严重的小企业36家，改造供热管网12.8公里、并网180万平方米。

　　畅通工程有序开展。改造城区街巷路百余条，东洲河口桥、石化大道和抚通、桓永高速公路建成通车。铺设农村“一事一议”村屯路591公里，工程量和建设速度全省第一。投资16.7亿元，新建和改造66千伏以上电网线路690公里。

　　清洁工程成效明显。整治街路17条、露天市场24个，取缔违规市场10个，打造管理示范路5条，街路人工清扫保洁率、生活垃圾清运率均达100%。

　　景观工程亮点频现。城区新增绿地197公顷，以浑河两岸、主干街路、旅游线路为重点的绿化水平大幅提高；亮化十里滨水公园、浑河南岸带状公园；完成月牙岛生态公园、高尔山公园续建工程，建成沈抚新城标志性景观——生命之环。建成抚顺县三块石旅游风景线，开工建设台沟湿地公园；改造完成清原河滨园、源中园休闲广场；绿化、亮化新宾苏子河北岸，启运大道成为辽宁省文化景观第一路。

　　（五）社会建设取得新进步

　　千方百计增加民生投入，尽心竭力办好惠民实事，各项社会事业跃上新台阶。

　　社会保障更加完善。加大扩面征缴力度，积极争取上级支持，保证了32.7万企业离退休人员养老金按时足额发放。提高城镇居民基本医疗保险补助标准，职工医保统筹报销比例由67.3%提至76.8%、位居全省第三。城市低保标准由每月330元提至390元，农村低保标准由每年1705元提至2400元，均居全省第五，惠及城乡16.6万人。

　　安居就业成效显著。拆迁改造旧城区106万平方米，竣工保障性住房、棚户区改造住房3659套，为百名农村单亲贫困母亲建房6千平方米，保障性安居工程建设得到省政府表扬。实施“暖房子”工程，清原县、新宾县分别完成180万平方米和30万平方米，1.9万户群众受益。普惠制就业培训4.8万人，登记失业率控制在4%以内。

　　扶贫帮困力度加大。城市贫困居民取暖救助4.1万户。健全从小学到大学的全程扶困助学体系，惠及学生2.4万人。慈善“助”字号工程资助困难群众2.1万人。减少农村低收入人口近8万人。投放中央财政贴息妇女小额担保贷款2.9亿元，位居全省第一，帮助6千户农村家庭走上了致富之路。

　　教育科技事业再上新台阶。新改扩建公办幼儿园16所，改造提升城区初中34所，新建改造农村学校76所。全域高分通过省“双高普九”评估验收，新抚区、顺城区通过省教育强县区预检。抚顺二中获北京大学自主招生校长实名推荐资格，新增国家级中职示范校1所。科技创新能力不断增强，我市被确定为全国产学研合作创新示范基地。

　　文化体育事业蓬勃发展。抚顺雷锋大剧院、煤矿博物馆、清原县体育馆建成使用，市图书馆、市民活动中心、沈抚新城文化馆、清原县满族博物馆即将竣工。新建体育健身广场270个、市民健身中心5个，高尔山健身登山步道达到国家标准。成功举办群众文化艺术节、读书节、登山节，群众参与度创历史之最。望花区煤精琥珀博物馆获“省科普教育基地”称号。与央视联办“放歌雷锋城”等全国性活动，百姓雷锋和道德模范层出不穷。

　　卫生事业全面推进。完成17个社区卫生服务中心和第二、第四、第五医院以及职业病防治院改扩建，改造完成标准化村卫生室200余所、县医院3所。农村免费孕前优生健康检查实现全覆盖。三县全面启动常年病人托管中心，规模水平位居全省前列。

　　 社会管理更加规范。推进社区规范化建设，269个城市社区全部实现网格化管理。强化社会治安防控，基本实现“无传销市”目标，荣获全国见义勇为城市奖。规范静态交通，整治特权车辆，增设监控抓拍系统近300处，交通秩序明显好转。实施食品药品安全专项整治，生产生活必需品市场价格基本稳定，政府应急管理逐步完善，防灾减灾能力全面提升。新建的国内一流的市殡仪馆即将交付使用。

　　其他各项工作都取得了新进展。

　　（六）建设人民满意政府迈出坚实步伐

　　把群众期盼作为工作追求，把人民满意作为检验标准，强化宗旨意识，提高服务水平。

　　坚持执政为民。牢固树立公仆意识，倾听群众呼声，将有限的财力更多投向与人民群众息息相关的社保、教育、医疗、道路、环保等领域。认真解决社会热点难点问题，市民来电来函诉求解决率达到97.2%，民心网、政风行风热线群众反映问题办结率达到98.1%、群众满意率达到98.5%。

　　提高执政能力。牢固树立服务意识，加强学习，科学决策，我市被评为全省依法行政示范市。强化责任制和督促检查，做到当日事当日毕，行政效能明显提升。坚持走进基层、走进项目、走进企业，抓实招商引资、抓实项目落地开工、抓实现有企业生产运营，有效破解发展难题。

　　主动接受监督。牢固树立自律意识，自觉接受市人大及其常委会的法律监督、工作监督和市政协的民主监督，办理人大代表建议363件、政协提案377件，办结率100%，解决率63.8%、为五年来最高。积极推进政务公开，及时公布重大决策和突发事件信息，保证群众的知情权、监督权和参与权。

　　尽管抚顺已经进入发展快车道，但作为全省最后一个摆脱“东北现象”的城市，抗风险能力还很脆弱。在肯定成绩的同时，我们更应正视发展中的问题。一是产业的丰厚度和完整度有待提高，城市化质量亟需提升。二是社会发展中深层次问题还没有得到根本解决，财政收入放缓与刚性支出增加的矛盾进一步加剧。三是民生期待不断增多，部分群众生活还很困难。四是农业农村基础设施建设还需加强，城市空气质量有待改善，生态环境保护任重道远。对此，我们将高度重视，认真加以解决。

　　各位代表，回顾过去，我们之所以能够在建设幸福抚顺的道路上披荆斩棘、加速前行，主要归功于省委、省政府和市委的正确领导，归功于市人大、市政协的大力支持，归功于全市230万人民的共同努力。

　　在此，我代表市政府，向全市各族人民，向人大代表、政协委员，向各民主党派、工商联，无党派人士、人民团体、离退休老同志，向驻抚部队、武警官兵、公安干警和中省直单位，向所有关心抚顺发展的朋友们，表示崇高的敬意和衷心的感谢！

　　二、未来五年奋斗目标及2013年重点工作

　　未来五年，是抚顺实现全面振兴的决胜期，也是全面建成小康社会的关键期。我们既要看到前进道路上的困难和挑战，更要看到面临的机遇和优势，义无反顾地承担起历史赋予我们的责任。当前，抚顺加快发展的态势已经形成，全市人民求发展的信心明显增强、谋振兴的氛围日渐浓厚。我们完全有条件、有能力，推动抚顺经济社会实现更高水平、更优质量、更快速度的发展，再攀高峰。

　　今后五年政府工作的总体要求是：全面贯彻落实党的十八大精神，以科学发展观为指导，以建设幸福美丽抚顺为目标，解放思想、改革开放、真抓实干、攻坚克难，坚定不移地实施“两城两带”、全域发展、多点支撑的发展战略，加快经济结构调整和发展方式转变，着力提升经济增长的质量和效益，显著提高人民生活水平，为全面建成小康社会奠定坚实基础。

　　经过五年奋斗，抚顺的发展将跃上一个新台阶。

　　 到那时，经济综合实力显著提升，发展方式明显转变，基本形成一产精、二产强、三产优的产业发展格局，现代化的沈抚新城、石化新城基本建成，县区实力明显增强，抚顺将成为全省重要的经济增长极。　到那时，城市更新和重点小城镇建设基本完成，城市功能更加完善，城乡一体化进程加快，现代交通体系基本形成，人居环境明显改善，城乡森林环抱、湿地相间、水系环绕、绿茵棋布，抚顺将成为国家生态园林城市。

　　 到那时，雷锋精神与城市精神相融合，社会文明程度明显提高，人民群众的权益和社会公平正义得到保障，人与人之间相互尊重、融洽相处，社会更加和谐稳定，抚顺将成为全国文明城市。

　　 到那时，社会就业更加充分，社会保障实现高水平的广覆盖，家庭财富显著增加，我们的孩子享有更好更优质的教育，人民物质生活更加富裕、精神生活更加丰富多彩，幸福感自豪感普遍增强。

　　 那时的抚顺，必将以幸福美丽城市的神奇魅力展现在世人面前，令人刮目相看，令人心驰神往，令人无限留恋。

　　各位代表，绘就五年宏伟蓝图必须从实现今年目标开始。当前的经济形势依然严峻复杂，我们必须从最难处着想，做最充分准备，稳中求进，进中求好，好中求快，实现有质量、有效益、可持续的增长。

　　2013年全市经济社会发展的主要预期目标是：地区生产总值增长12%，公共财政预算收入增长15%，全社会固定资产投资增长25%，社会消费品零售总额增长15%，城市居民人均可支配收入增长14%，农民人均纯收入增长15%。

　　我们深知，敬业才能成就事业，尽责才能赢得民心。有了责任心，就会时刻保持危机感和紧迫感，恪尽职守，永不懈怠；就不会对竞争熟视无睹、对差距无动于衷、对落后麻木不仁。各级政府、每一个公务员都要敢于负责、能够负责、对人民负责，从现在做起，从自身做起，从细节做起，从能够做的事情做起，把各项目标任务工程化、具体化，不断夺取经济和社会发展的新胜利！

　　今年，我们将重点做好八个方面工作。

　　（一）坚持工业强市，着力发展实体经济

　　以新型工业化为引领，做大总量，提升质量，为稳增长提供重要支撑。

　　抓生产运营，稳定存量。继续坚持走进企业，解决困难。帮助企业协调生产要素，开拓市场，确保现有企业满负荷生产；围绕装备制造、化工及精细化工等重点产业，确保竣工企业达产达效；跟踪服务50个重点项目，确保新建企业竣工投产。

　　抓产业集群，扩大增量。加大招商引资力度，加快完善园区基础设施，积极推进公共服务平台建设，壮大工程机械及制品、化工新材料两大千亿产业集群规模，叫响县区10个百亿产业集群品牌。

　　抓自主创新，提升发展质量。大力实施创新驱动战略，以利用辽宁省鼓励研发国内首台（套）装备政策为切入点，抓好一批科技创新工程和技术改造升级项目。推进产学研合作，引进海外先进技术，提高企业核心竞争力。全年高新技术产业增加值增长15%。

　　抓工业地产，加快中小企业集聚。引进知名工业地产企业，推进重点项目建设，全年开工建设标准厂房200万平方米，建成投用100万平方米。加大扶持力度，重点培育50户创新型中小企业，力争进入全省年营业收入超10亿元企业行列。

　　（二）以旅游业为牵动，大力发展服务业

　　现阶段，要坚持以发展商贸流通业为重点、旅游业为突破口，留住本地消费，吸引外地消费，尽快将服务业做大做优。全年服务业增加值增速不低于地区生产总值增速。

　　打造旅游目的地城市。依托丰富的资源和广阔的市场，把抚顺建设成为旅游强市。进一步完善水、电、路等配套基础设施，积极推进重点旅游项目建设，加快星级酒店、温泉小镇、休闲山庄等旅游地产开发。做大沈抚新城娱乐文化、清原生态、新宾兴京文化、抚顺县三块石休闲等四个旅游集聚区，规划建设相匹配的四条旅游精品线路。坚持常规游与节日游相结合，以全运会为契机，继续举办满族风情节、冰雪节、戏水节，包装策划春季赏花、夏季戏水、秋季采摘、冬季滑雪等主题活动，推出旅游品牌，提升抚顺旅游知名度。

　　 巩固提升商贸服务业。科学规划商业布局，完善城市功能，提供多层次消费服务。推动城区经济健康发展，围绕六大商贸服务业集聚区，加快城市综合体项目建设，确保在建商业项目建成运营，鼓励支持原有大型商贸企业多元化发展、连锁化经营，打造榆林采沉区等一批承载能力大、带动作用强、辐射范围广的专业批发市场。加快发展夜经济，培育新的消费热点。促进楼宇经济、总部经济和金融服务、文化创意、会展等现代服务业发展。加快发展生产性服务业，提高产业配套能力。

　　（三）以开发生态农业为重点，加快发展农村经济

　　坚持生态立县，将特色农业、沟域经济与生态建设有机结合，构建“一县一业”、“一乡一品”特色农业发展格局。

　　打造特色产业基地。稳定粮食和主要农畜产品产量，依托“一县一业”主导产业，重点打造食用菌、山野菜、中药材、百合花四大特色产业基地，把新宾县建成全省香菇产业第一县，把抚顺县建成全省单片黑木耳产业第一县，把清原县建成东方百合种球繁育生产基地和全省最大的中药材生产基地。

　　大力发展沟域经济。坚持生态优先、农游结合、精品特色、富农惠农的原则，继续深化集体林权制度改革，促进现代农业和林下经济加快发展。大力扶持龙头企业，提升产业化经营水平。培育一批功能性农产品，打造一批地理标志产品，提高农产品附加值，促进农民增收。

　　（四）加快基础设施建设，推动全域多点发展

　　 统筹城乡与区域发展，坚持深耕城市化和新型城镇化齐头并进，继续保持百亿元城建投资规模，建设功能完备、环境优美的宜居城乡。 

　　继续举全市之力建设沈抚新城。坚持“三给”政策，打造国际知名、全国一流的现代高端城市。加快推进以交通、金融、教育、卫生、户籍和社保为重点的沈抚同城化步伐。进一步完善城市功能，打造标志景观，建成玉露潭公园、生命之环广场，完成幼儿园和中小学建设，投入使用市中医院、文化中心、金融大厦，继续拓展旅游项目，完善旅游配套设施，建成营业一批城市综合体，打造沈阳经济区休闲娱乐购物新天地。

　　加快建设石化新城。坚持工业园区建设与城市更新改造并举，建设现代化城区。依托石化产业优势，打造化工及精细化工产业基地。推进内陆港和工业管廊、污水处理、集中供热等基础设施建设，投入运行东洲、哈达、章党和海新污水处理厂，萨尔浒大道、海赋通道、海赋大桥、章党大桥年内全线通车，建成好运角生态公园。加速推进以东洲大街和绥化路为轴线的城市区域中心建设。

　　全面改造老城区。结合城区“退二进三”，加快城市更新，统一规划，成片开发，继续保持百万平方米以上拆迁规模，尽快改变老城区面貌。坚持绿色生态与自然水系景观有机结合，做好水系和绿化文章，逐步将抚顺建成水在城中、城在绿中、人在景中的水城和森林城市。全面改造南站地区，引入浑河水系，改造63条街巷路，打造集商业、旅游观光为一体的地下水城，建设城市客厅。整治华山周边地区，加快抚顺发电厂、石油一厂和水泥厂搬迁，启动建设具有休闲娱乐、文化展示功能的工业遗址公园。加快丹东路和雷锋路沿线更新改造，推进重点服务业项目建设，打造区域性商贸核心区。加快推进新华地区、葛布地区改造开发，与城东新区尽快形成商贸服务业格局。

　　 加快建设县城和小城镇。坚持以城带乡，完善基础设施，改善人居环境，提升承载能力，大力推进新型城镇化，实现由“乡”到“城”的转变。清原县城重点是提升档次与水平，加快推进“一口、一线、一区”建设。新宾县城重点是拓展发展空间，开发建设西部新城，启动热源改造工程。抚顺县以石文镇获批全国发展和改革试点镇为契机，加快小城镇建设。三县围绕3条旅游精品线路，积极推进沿线重点小城镇规划建设。

　　完善城市基础设施。新建改造66千伏及以上电网线路768公里，新增66千伏及以上变电容量159万千伏安。建设城东三期道路、供排水管网等基础设施。三年内完成气化抚顺工程，今年全面改造望花和虎万地区燃气管网，两地居民年内用上管输天然气。

　　（五）扩大对外开放，加快项目建设步伐

　　坚定不移对外开放，以更加开放的姿态、更加优质的环境、更加高效的服务吸引投资。继续开展项目年活动，以项目大建设推动经济大发展。

　　 强化招商引资，积极向上争取。完善招商组织协调和落实激励机制，充分调动招商主体的积极性，发挥现有企业、商会、社团组织作用，立足区位优势、资源特色、产业基础，创新招商方式，实现招商引资新突破。深入研究国家政策导向，做好前期准备，争取在扩大内需、产业发展、民生改善、资源补偿、生态恢复等方面得到更大的支持。

　　强化要素保障，加快项目落地。坚持集约节约用地，用足用活政策，保障项目用地。加大金融信贷对地方经济的支持，搭建银企对接平台，鼓励并用好社会资本。完善机制，营造环境，吸引、留住优秀人才。强化首问负责，扎实做好立项、环评、供电、道路、消防等审批和服务，加快项目落地建设。 

　　（六）加大环境保护力度，突出抓好生态文明建设

　　强调绿色发展、循环发展，在保护中发展，让生态环境更加优美宜居、人民生活更加舒心惬意。

　　巩固提升绿水工程。确保新建污水处理厂、垃圾收集处理设施稳定运行，实现污水达标排放。实施浑河、社河和苏子河生态安全防护工程。整治农村中小河流，治理前安河、大苏河等重点河道。

　　深入推进青山工程。加大退耕还林力度，加快矿山生态恢复治理，突出整治公路沿线和城市周边损坏山体，严厉打击破坏森林资源的违法行为，重点抓好大伙房水库周边山体生态治理，打造5条绿化精品线路。

　　继续实施蓝天工程。推进集中供热，落实“一县一热源”，加快抚矿集团两台30万千瓦供热机组建设和中电投辽宁发电公司两台35万千瓦供热机组改造，并网面积400万平方米。推进落后产能提标淘汰，严格监管重污染企业，坚决关停限期未达标企业。推进重点区域污染整治，狠抓城市扬尘治理，建立大气监测预警系统和污染减排考核机制，严厉打击违法排污行为。

　　（七）保障改善民生，构建和谐社会

　　顺应群众期待，将发展成果更多更公平地惠及全市人民。

　　 学有所教。普及规范学前教育，均衡发展义务教育，鼓励支持民办教育，合理布局高中、中职和高等教育。继续加大财政投入，重点改造提升高中和中职学校。选拔一流校长、培养一流教师、建设一流学校，实现办学硬件条件与教育教学水平同步提升，让每个孩子都能公平享受优质教育。

　　 业有所就。加强职业技能培训，鼓励自主创业，扶持中小微企业，落实减免税费、贷款贴息等扶持政策，加快发展旅游、商贸等富民产业，实现更加稳定、更高质量的就业。全年城镇实名制就业9.5万人、零就业家庭动态为零。

　　 病有所医。巩固完善新农合制度，全面推行城乡居民大病保险，巩固农村三级医疗卫生服务体系，贴息鼓励各级各类医院全面改善就医条件。

　　老有所养。全力筹措社保资金，确保企业离退休人员养老金按时足额发放。鼓励社会力量兴办养老机构，完成望花、顺城社会福利中心建设。

　　困有所助。继续提高城乡低保、孤儿养育、农村五保供养标准和重点优抚对象救助标准，保障困难群众基本生活。大力发展慈善事业，维护老龄、妇女儿童和残疾人等群体的合法权益。

　　住有所居。加强保障性安居工程建设，继续推进棚户区改造和“暖房子”工程，加强物业管理，着手解决弃管小区问题，改造老旧房屋供水设施２万户，解决农村5.3万人安全饮水问题。

　　貌有所变。将城市建设成果继续向村屯和社区延伸，重点改造街巷路200余条，优先发展公共交通，延伸调整部分公交线路，维修公交站牌，更新出租车。用好省“一事一议”奖补政策，整合财政资金，继续开展农村环境集中整治，新建农村道路400公里，整体推进广场建设、村屯绿化、边沟治理和农厕改造等工作。

　　乱有所管。加快“数字抚顺”建设，创新社会管理机制，强化城市细节管理，将严格管理与正面引导相结合，提高群众爱护城市的自觉性和主动性。继续规范交通秩序，实现电子监控全域覆盖；继续推进清洁工程，重拳治乱，净化环境，共建美好家园。

　　民有所乐。加快群众文化场馆建设，免费开放各类公共文化设施。推进重点文化惠民工程，将群众性节庆活动常态化，打造抚顺特色文化品牌。加快建设体育主题公园和县区市民健身中心，广泛开展群众喜闻乐见的体育活动，扎实做好承办“十二运”的各项筹备工作，让全市人民充分享受“十二运”带来的体育盛宴。

　　安有所保。扎实推进“平安抚顺”建设，加大社会治安防控力度，畅通信访诉求渠道，强化法律援助，维护人民群众合法权益。全面落实安全生产责任，严格食品药品监管。完善防灾减灾体系，提高应急处置能力，保障人民群众生命财产安全。

　　继续做好其他各项工作。

　　各位代表，民生连着民心、连着发展、连着和谐。我们要坚持发展经济和改善民生相结合、富民与强市相统一，心系百姓，为民担当，矢志不渝地为人民群众做好事、办实事、解难事，努力让抚顺人民生活得更加舒心、更加体面、更有尊严！

　　（八）增强公仆意识，努力建设人民满意的服务型政府

　　以人为本，为民谋利。不断增强与人民群众的血肉联系，自觉把执政为民融入到工作思路里、体现在政策措施中、落实到具体工作上。对群众关心的事，定一条办一条，条条算数；向群众承诺的事，说一项做一项，项项兑现；与群众利益发生冲突的事，有一件改一件，件件纠正。

　　高效实干，雷厉风行。继续“三抓实、三走进”，多到条件差、问题多、工作难度大、人民群众呼声高的地方，解决实际问题。作出的决策，执行坚决，贯彻有力；定下的工作，逐件办理，步步扎实；遇到的问题，逐个解决，不留遗憾。以速度抢机遇，做到事不过夜，立说立行。

　　凝聚力量，攻坚克难。更加注重权利公平、机会公平、规则公平，兼顾各方利益，调动各方积极性，凝聚共识，赢得支持。始终保持敢为人先的勇气，解放思想，勇于开拓。始终保持昂扬向上的朝气，把精力集中在想干事上、本领体现在会干事上、目标锁定在干成事上。始终保持争创一流的锐气，在扬长避短中创造优势，在超越自我中再创佳绩。

　　依法行政， 保持清廉。自觉接受人大及其常委会的法律监督、工作监督和政协的民主监督，充分听取民主党派、工商联、无党派人士和各人民团体的意见建议，广泛接受新闻舆论和社会公众监督。强化行政监察和审计监督，严肃查处各类违纪违法案件，珍惜并用好纳税人的每一分钱，努力建设清廉政府、节约机关。

　　各位代表，时代赋予重任，奋斗铸就辉煌。我们要倍加珍惜来之不易的发展态势，倍加珍惜团结和谐的大好局面，以更加坚定的信念、更加务实的作风，同心同德，锐意进取，为全面建成幸福美丽抚顺而努力奋斗！
